# Efficacy of Wide-Awake Local Anesthesia No Tourniquet (WALANT) Versus Bier’s Block in the Internal Fixation of Unstable Distal Radius Fractures: A Single-Center Experience

**DOI:** 10.7759/cureus.98826

**Published:** 2025-12-09

**Authors:** Grigorios Kastanis, Constantinos Chaniotakis, Mikela-Rafaella Siligardou, Petros Kapsetakis, Nikolaos Ritzakis, Chrysostomos Tsatsoulas, Ioannis Ktistakis, Ioannis M Stavrakakis

**Affiliations:** 1 Department of Orthopedics, Venizeleio General Hospital, Heraklion, GRC

**Keywords:** biers block, distal radius fracture, internal fixation, palmar locking plate, walant

## Abstract

Introduction and aim: Unstable distal radius fractures typically necessitate surgical management through open reduction and internal fixation (ORIF). There are significant considerations and challenges regarding the selection of the anesthetic modality employed for the surgical management of these fractures. Recently, the wide-awake local anesthesia no tourniquet (WALANT) technique has been increasingly used for hand and wrist procedures, demonstrating excellent outcomes and fewer complications compared to general or regional anesthesia. This study aimed to compare the efficacy of the WALANT technique with Bier’s block anesthesia in the fixation of unstable distal radius fractures.

Materials and methods: Patient demographics, mechanism of injury, fracture type, and anesthesia type (Bier’s block or WALANT) were recorded. Our study constitutes a retrospective comparative analysis between the two types of anesthesia employed. All fractures were fixed through the extensor carpi radialis approach using a palmar locking plate. Surgeries were performed within 10 days of injury (range: one to 15 days). Pain levels during injection (WALANT group), intraoperatively, and postoperatively were assessed using the visual analog scale (VAS). Additional parameters included duration of surgery, analgesic use, and preoperative anxiety using the Amsterdam Preoperative Anxiety and Information Scale (APAIS).

Results: Fifty-one patients (mean age: 57.9 years; range: 41-73) underwent ORIF - 26 with Bier’s block and 25 with WALANT. Mean intraoperative pain in the WALANT group was 1.48/10, with postoperative VAS 1/10, compared to 6/10 in the Bier’s block group. APAIS scores were similar between groups (4.45 in WALANT versus 4.58 in Bier’s block). WALANT patients were discharged the same day, while those with Bier’s block were discharged the following day. Analgesic use was shorter in the WALANT group (2.3 days versus 5.7 days). Surgery duration was slightly longer with WALANT (60.1 versus 54.2 min).

Conclusion: The use of WALANT offered lower pain scores, reduced analgesic use, and same-day discharge. Therefore, WALANT is recommended as the preferred anesthetic technique for unstable distal radius fracture fixation.

## Introduction

Distal radius fractures (DRFs) are the most frequent upper-limb injuries encountered in the emergency department. These fractures demonstrate a bimodal age distribution as follows: in younger adults, they are typically associated with high-energy trauma, whereas in the elderly population, they are often related to osteoporotic bone [1]. Conservative management with cast immobilization for unstable DRFs frequently fails to maintain fracture reduction until consolidation, with reported failure rates exceeding 50% [2]. In recent years, open reduction and internal fixation (ORIF) with a volar locking plate has been widely adopted, as it provides stable anatomical reduction and facilitates early mobilization, thereby improving functional recovery [3,4].

Traditionally, ORIF of DRFs is performed under tourniquet-assisted conditions to minimize intraoperative bleeding. However, prolonged surgery and tourniquet application can result in significant patient discomfort, often necessitating general anesthesia (GA) or regional anesthesia, such as Bier’s or brachial plexus block [5]. GA carries an increased risk of perioperative complications, particularly in patients with comorbidities such as cardiopulmonary disease, whereas regional blocks require technical expertise and specialized equipment [6].

In 2005, Lalonde et al. introduced the wide-awake local anesthesia no tourniquet (WALANT) technique, which involves local infiltration of lidocaine and epinephrine, permitting surgery without sedation or tourniquet use [7]. WALANT has been successfully applied in a range of hand procedures, including carpal tunnel release, trigger finger release, tendon repair or transfer, wrist arthroscopy for triangular fibrocartilage complex repair, and internal fixation or implant removal for metacarpal and phalangeal fractures [8-12]. The technique is safe, cost-effective, and allows intraoperative assessment of the active range of motion, potentially enhancing postoperative rehabilitation. Recently, WALANT has been applied to distal radius fracture fixation, with studies reporting comparable or superior outcomes in reduction quality, functional recovery, and patient satisfaction compared with GA or regional anesthesia [2,4,5,13-15].

The objective of this study is to compare the efficacy of WALANT versus Bier’s block in the fixation of unstable DRFs, evaluating intraoperative and postoperative pain using the visual analog scale (VAS), operative time, duration of analgesic use, preoperative anxiety assessed by the Amsterdam Preoperative Anxiety and Information Scale (APAIS), and six-month postoperative functional outcomes and range of motion measured by the Quick Disabilities of the Arm, Shoulder, and Hand (QuickDASH) score [14].

## Materials and methods

This study was conducted at a level II trauma center (Department of Orthopedics, Venizeleio General Hospital) between July 2023 and December 2024. Our study constitutes a retrospective comparative analysis between the two types of anesthesia employed. All adult patients who sustained a DRF and underwent ORIF under either Bier’s block (BB) or WALANT anesthesia during this period were included in the study. The study protocol was approved by the Institutional Ethics Committee, and written informed consent was obtained from all participants prior to inclusion. The statistical analysis was performed using the SPSS software, version 24.0 (Armonk, NY: IBM Corp.). The statistical tests used were χ², Student's t-test, and ANOVA.

The indications for surgical treatment were displaced distal radius fractures with an intra-articular step greater than 2 mm, radial shortening exceeding 2 mm, dorsal tilt greater than 15°, incongruence of the distal radioulnar joint, or bilateral DRFs. Fractures were classified according to the AO Foundation/Orthopedic Trauma Association (AO/OTA) classification system. All surgeries were performed by the senior author within 10 days of injury (range: one to 15 days). Inclusion and exclusion criteria are summarized in Table 1. Patients’ demographics, mechanism of injury, type of fracture, type of anesthesia (BB or WALANT), duration of surgery, intraoperative and postoperative VAS score, and APAIS score were reported (Table 2). The normal distribution of all continuous variables was confirmed using the Kolmogorov-Smirnov test. The mean duration of surgery, mean preoperative and postoperative VAS scores, mean duration of postoperative analgesia, and APAIS score were compared between the group of patients who received BB and the group of patients who received WALANT using the Student’s t-test for independent variables.

**Table 1:** Inclusion and exclusion criteria.

Inclusion criteria	Exclusion criteria
Age >18 years	Age <18 years
Acute isolated closed or open fractures without soft-tissue defects	History of scleroderma, Raynaud’s phenomenon, or vasculitis
Absence of cognitive impairment (e.g., dementia)	Corrective osteotomies for malunions
A minimum follow-up of six months	Open fractures with extensive soft-tissue injury
-	Polytrauma patients
-	Concomitant vascular injury of the affected hand.

**Table 2:** Patients’ demographics - type of anesthesia, type of fracture, and tests used for analysis. WALANT: wide-awake local anesthesia no tourniquet

Variables	WALANT (n=25)	Bier’s block (n=26)	Statistical test	t-value, χ², F-value	p-Value
Gender (male/female)	9/16 (40%/60%)	12/14 (46.2%/53.8%)	χ²	χ²=0.29	0.59
Mean age (years)	62.9 (45-73)	56.6 (41-70)	Student t-test	t-value 2.14	0.09
Hand involved (right/left)	18/7 (72%/28%)	17/9 (65.4%/34.6%)	χ²	χ²​​​​​​​=0.32	0.56
Dominant hand (right/left)	17/8 (68%/32%)	19/7 (73.1%/26.9%)	χ²	χ²​​​​​​​=0.102	0.75
Type of fracture - AO classification	A3: 4 (16%)	A3: 4 (15.4%)	Anova	F-value=0.26	0.97
	B2: 3 (12%)	B2: 4 (15.4%)			
	B3: 8 (32%)	B3: 8 (30.8%)			
	C1: 5 (20%)	C1: 6 (23%)			
	C2: 5 (20%)	C2: 4 (15.4%)			
Bilateral	3 (12%)	4 (15%)	χ²	χ²​​​​​​​=0.46	0.49
Ulnar styloid fracture	13	12	χ²	χ²​​​​​​​=0.102	0.75
Open fracture Gustilo and Anderson	I: 2 (8%)	I: 1 (3.9%)	χ²	χ²​​​​​​​=2.04	0.60
	II: 1 (4%)	II: 0			
Complementary anesthesia	0 (0%)	4 (15.4%)	χ²	χ²​​​​​​​=4.23	0.04

Anesthesia

WALANT Procedure

**Figure 1:**
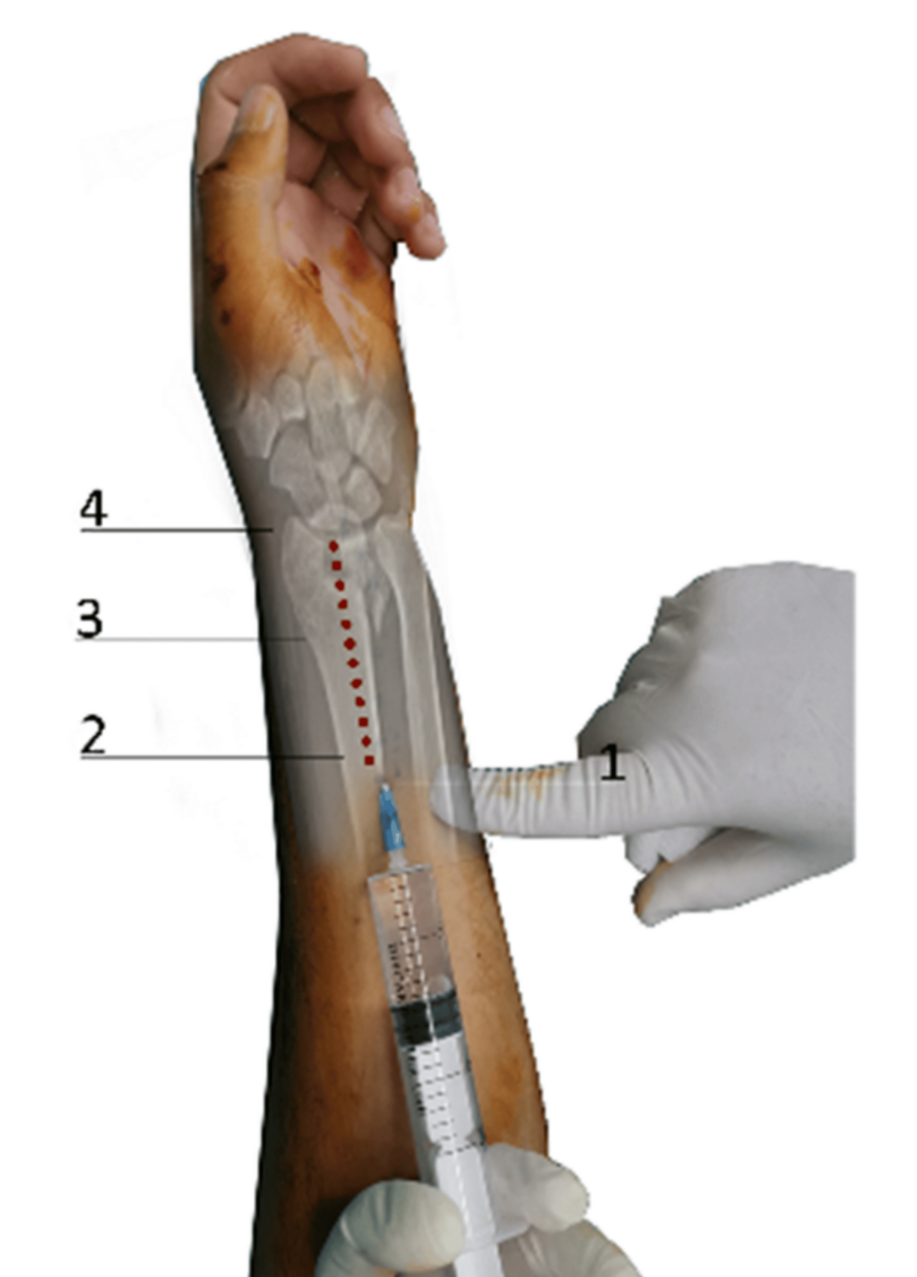
Schematic description of WALANT. (1) Infiltration of 10 mL of tumescent at the surgical approaches; (2) infiltration of periosteum at the radial border of the radius (proximal end of the plate), 4 mL and 2 mL at the volar and dorsal side of the bone; (3) 10 mL tumescent at the middle side of the plate; and (4) last 10 mL at the distal radius. WALANT: wide-awake local anesthesia no tourniquet This image is original to this study and was created by Mr. Grigorios Kastanis.

We prepared an analgesic-hemostatic mixture of 10 cc of premixed 1% lidocaine (4 mL), 1:100,000 adrenaline (1 mL), normal saline (4 mL), and 1 mL of 8.4% sodium bicarbonate according to the Lalonde technique [8]. To decrease injection pain, a 27-gauge needle was used, and the injection was given slowly according to the instructions by Hamelin et al. [16]. Firstly, a total of 10 mL was infiltrated subcutaneously along the surgical incision (extended flexor carpi radialis approach) from proximal to distal (Figure 1). The second step was to infiltrate a deeper layer of local anesthesia at the proximal part of the radial border of the distal radius. Approximately 4 mL was infiltrated in the periosteum of the radial border and 2 mL on the dorsal and volar surface of the distal radius. We continued with the same mode, infiltrating 10 mL into the middle aspect of the plate location and another 10 mL into the distal radius. After incising the pronator quadratus muscle, we infiltrated 2 mL at the volar side of the distal radioulnar joint. In case of a concomitant fracture of the ulnar styloid, we infiltrated 4 mL into the distal ulna. The total mixture used was 40 mL.

Bier's Block Procedure

The technique was performed in the operating theatre by the anesthesiology personnel. A pneumatic tourniquet was inflated to an average of 250 mmHg (range: 220-270 mmHg) depending on the patient's blood pressure. An intravenous 40 mL mixture of lidocaine 2% 2.5 mg/kg, 5 mL ropivacaine 0.75%, meperidine 1 mg/kg, dexamethasone 8 mg, ondansetron 8 mg, magnesium 25% 20 mg/kg, and N/S 0.9% was given afterwards. Sensory and motor blockage was obtained after 10 min from the injection.

Surgical Procedure

The patients were placed in the supine position and were monitored closely. In the WALANT group, the operation started at least 25 min after the local anesthesia injections. In the BB group, the operation started approximately 10 min after the anesthesia. The extended flexor carpi radialis approach described by Orbay et al. was performed [15].

**Figure 2:**
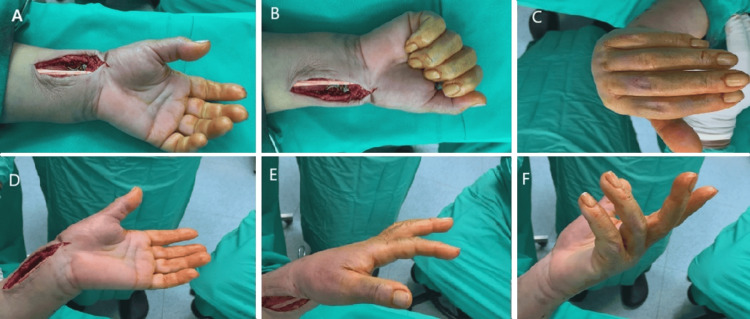
Intraoperative assessment of osteosynthesis stability through full wrist range of motion. The images show (A) finger extension, (B) finger flexion, (C) wrist flexion, (D) wrist extension, (E) forearm pronation, and (F) forearm supination.

In the WALANT group, patients were asked to perform active range of motion (ROM) of the involved wrist to assess the stability of the osteosynthesis (Figures 2A-2F). In the BB group, fixation stability was instead assessed by passive wrist ROM performed by the surgeon. Finally, in both groups, examination with C-arm intensifier was performed to check the final osteosynthesis and screws length.

Postoperatively, a volar splint was applied to all patients to control swelling and pain and enhance soft-tissue healing. Postoperative analgesia included paracetamol (1 g orally every 6 h) and ibuprofen (400 mg orally every 6 h). The stitches were removed in two weeks. Splint immobilization was maintained for two to four weeks more, depending on the comminution of the fracture and the quality of fixation. A standard rehabilitation protocol program from the first postoperative day was applied in all patients, starting from the adjacent joints to the wrist. Active-assisted exercises and ROM of the wrist were initiated after the immobilization phase.

## Results

Fifty-one patients (21 men and 30 women) with a mean age of 57.9 years were retrieved from the institutional patient registry. Twenty-five patients underwent the WALANT technique (first group), and 26 patients underwent the BB technique (second group). Patients’ demographics and fracture types were similar between the two groups. The main injury mechanism was a fall from standing height onto an outstretched hand (Table 3). According to the AO/OTA classification system, the following fracture types were recorded: 23-A3 = eight cases (15.7%), 23-B2=seven cases (13.7%), 23-B3=16 cases (31.4%), 23-C1=11 cases (21.6%), and 23-C3=nine cases (17.6%). Three patients sustained an open grade I DRF, and one patient sustained an open grade II DRF, according to the Gustilo-Anderson classification.

**Table 3:** Cause of distal radius fracture.

Cause	Patients, n (%)
Fall from standing height	37 (72.5)
Sport injuries	4 (7.9%)
Vehicle accident	8 (15.7%)
Industrial injuries	2 (3.9%)

**Figure 3:**
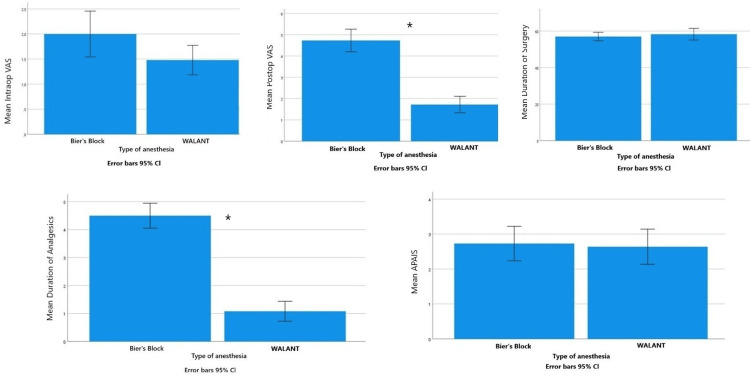
Intraoperative VAS score (p=0.055, NSS), postoperative VAS score (*p<0.001), duration of surgery (p=0.507, NSS), duration of analgesia (*p<0.001), APAIS score (p-value: 0.792, NSS) of the WALANT and BB group of patients. *P<0.001 was statistically significant. NSS: no statistically significant; BB: Bier's block; WALANT: wide-awake local anesthesia no tourniquet; APAIS: Amsterdam Preoperative Anxiety and Information Scale; VAS: visual analog scale

The mean duration of surgery for the BB group and the WALANT group was 57.04 min (95% CI: 54.72-59.36) and 58.32 min (95% CI: 55.12-61.52), respectively (Figure 3). No statistically significant difference was identified between the two groups (p=0.507).

The mean intraoperative VAS score was 2 (95% CI: 1.54-2.46) for the BB group and 1.48 (95% CI: 1.19-1.77) for the WALANT group (p=0.055). The mean postoperative VAS score was 4.74 (95% CI: 4.20-5.26) for the BB group and 1.72 (95% CI: 1.33-2.11) for the WALANT group. This difference was found to be highly statistically significant (p<0.001). In the BB group, the mean duration of postoperative analgesic use was 4.5 days (95% CI: 4.05-4.95), whereas in the WALANT group it was 1.08 days (95% CI: 0.72-1.44) (p<0.001) (Figure 3).

The APAIS score was recorded before the administration of anesthesia. The mean APAIS score was 2.73 (95% CI: 2.24-3.22) for the BB group and 2.64 (95% CI: 2.14-3.14) for the WALANT group. No statistically significant difference was found between the two groups regarding the APAIS score (p=0.792) (Figure 3). APAIS ≥11 is high anxiety.

## Discussion

DRFs account for approximately 44% of all upper-limb fractures and represent the second most common fracture type in adults aged 18-50 years, constituting up to 25% of all fractures overall [15-17]. The treatment of unstable DRFs typically involves open reduction and internal fixation with a volar locking plate to allow early range of motion, prevent wrist dysfunction, and facilitate a rapid return to preinjury activities and employment [2,18].

Traditionally, such surgical procedures are performed under regional blocks or general anesthesia. However, these anesthetic techniques are associated with potential complications, including inadvertent intravascular injection and neurotoxicity of anesthetic agents [7]. A pneumatic tourniquet is often used in these cases to control bleeding and provide a bloodless surgical field [19]. Maury and Roy reported tourniquet tolerance among healthy volunteers, with mean tolerance times of 25 min for the forearm and 18 min for the upper arm, accompanied by transient pain and paresthesia [20]. Odinsson and Finsen reported a 0.05% incidence of neurological complications associated with tourniquet use during upper-limb surgery, with 87% of affected patients achieving full recovery within six months [21]. Additional studies have highlighted further tourniquet-related complications, including post-tourniquet thrombosis, bleeding, postoperative pain, skin blisters or burns, ischemia-reperfusion injury, and neuromuscular damage, particularly when the duration of application exceeds 20 min. These complications may further hinder postoperative rehabilitation [12].

The long-standing belief that the use of epinephrine in hand surgery can lead to irreversible ischemic complications, such as gangrene or necrosis, was refuted by Lalonde et al. in a multicenter prospective study involving 3,110 consecutive patients [7]. Based on these findings, Lalonde et al. introduced the WALANT technique [7]. This technique is based on two key principles. The first is the tumescent injection of a large volume of low-concentration lidocaine combined with epinephrine, which provides effective anesthesia extending at least 2 cm beyond the surgical area, while epinephrine-induced vasoconstriction minimizes intraoperative bleeding [22]. The second principle is that, when administered correctly, this tumescent injection is nearly painless, with the patient experiencing only the initial puncture from a fine (30G) needle [22].

Although historical reports of digital necrosis were attributed to the combined use of procaine and epinephrine, concerns persist regarding epinephrine-induced vasoconstriction in the digits. Nodwell and Lalonde demonstrated that the “white finger” phenomenon can be completely reversed by subcutaneous injection of 1 mg of phentolamine diluted in 220 mL of saline at the site of epinephrine infiltration [23]. In our study, no complications, such as digital necrosis or cyanosis, were observed following the use of epinephrine.

Recently, the WALANT technique has been increasingly utilized in various hand surgeries, particularly in soft-tissue repair and fracture fixation procedures [12,23]. The advantages of this method include the avoidance of tourniquet-related complications, such as postoperative discomfort, muscle ischemia or necrosis, and nerve injury [2,4]. It also eliminates the need for sedation, allowing for faster recovery and reducing complications associated with general or regional anesthesia, such as nausea, vomiting, and anesthesia-related risks in elderly patients with multiple comorbidities, while shortening postoperative recovery time. Furthermore, it does not require preoperative fasting, minimizing glycemic fluctuations in diabetic patients [6,8,10]. The technique also contributes to reduced healthcare costs by decreasing the need for preoperative investigations, shortening hospitalization, lowering opioid consumption, and conserving medical resources. Finally, intraoperative patient cooperation enables the surgeon to assess fixation stability or tendon repair in real time and make necessary adjustments during the procedure [12,24,25].

The application of WALANT for distal radius fracture plating has been reported only in recent years [26,27]. Ahmad et al. first described the use of WALANT in a single case of DRF, followed by Orbach et al., who presented five cases. Both studies concluded that WALANT provides a simple and safe alternative to conventional anesthetic methods for DRF fixation [2,26]. Huang et al. compared GA with WALANT for DRF fixation and found that although intraoperative blood loss was lower in the GA group, patients experienced more postoperative pain, while functional outcomes were comparable between groups. No complications were reported with either technique, confirming that WALANT is a feasible and effective anesthetic approach for distal radius plating [4].

Abitbol et al. compared WALANT with regional anesthesia and reported shorter postoperative analgesic use in the WALANT group [28]. In our study, both intraoperative (1.48 versus 2.0) and postoperative (1.72 versus 4.74) VAS scores were lower in the WALANT group compared with the BB group; however, a statistically significant difference was observed in the postoperative VAS score. Similarly, the mean duration of postoperative analgesic use was significantly shorter in the WALANT group (1.08 versus 4.50 days; p<0.001).

Dukan et al. noted that the WALANT technique may help prevent both early and delayed complications, such as median nerve injury and delayed rupture of the flexor pollicis longus (FPL) tendon [25]. Plates positioned at or distal to the watershed line are at greater risk of impinging on the FPL tendon, with reported rupture rates ranging from 1% to 12% [27]. Under WALANT, median nerve motor function and FPL tendon excursion can be actively evaluated intraoperatively. In our study, real-time patient cooperation (active thumb motion) enabled the surgeon to assess tendon gliding when the plate was positioned near the watershed line. In contrast, in the BB group, tendon mobility could be assessed only passively.

Preoperative anxiety was assessed using the APAIS scale, which showed no statistically significant difference between the two groups (BB group: 2.73; WALANT group: 2.64). Adequate preoperative counseling is essential, as patient anxiety can negatively influence intraoperative cooperation; an APAIS score above 11 may be considered a relative contraindication to the WALANT technique [28].

These savings derive from the elimination of preoperative tests and intraoperative sedation, reduced analgesic use, shorter hospital stays, and earlier return to work [4,6,25,28]. Rhee et al. also reported substantial cost savings - up to 85% - for comparable hand procedures performed under WALANT compared with other anesthetic techniques [29].

Lawand et al. reported in a systematic review that the complication rate associated with the WALANT technique is very low (1.7%), with only minor superficial infections and extremely rare cases of allergic reactions to lidocaine or epinephrine [30]. Similarly, Tahir et al. noted that the WALANT procedure should be avoided in patients with needle phobia, peripheral vascular disease, active infection, bleeding tendencies, abnormal coagulation profiles, or known hypersensitivity to lidocaine [6]. In the present study, all such conditions were included among the exclusion criteria.

The main limitations of this study are the relatively small sample size, the lack of randomization and blinding, the single-center design, the short follow-up, and the retrospective design. Therefore, further studies with larger cohorts and longer follow-up are required to confirm these findings and to more comprehensively evaluate the potential benefits of the WALANT technique in distal radius fracture fixation.

## Conclusions

Our results indicate that the WALANT technique is a safe and effective anesthetic method for ORIF of distal radius fractures. It is an alternative to traditional anesthetic techniques, particularly for patients with significant comorbidities, as it avoids the potential complications associated with general or regional anesthesia. In addition to its other advantages, such as cost-effectiveness, reduced preoperative preparation time, shorter postoperative hospitalization, and decreased pain, the main benefit of WALANT in ORIF of distal radius fractures is the opportunity for intraoperative active wrist motion. This enables real-time assessment of fixation stability and helps prevent postoperative complications related to implant impingement.
